# Recurrent Anastomotic Leak After Hartmann Reversal: Successful Management With Vacuum-Assisted Endoscopic Drainage and Diversion

**DOI:** 10.14309/crj.0000000000001976

**Published:** 2026-02-04

**Authors:** Ali Ghanem Al Masad, Omar Reda Abdelmaksoud

**Affiliations:** 1 Gastroenterology & Hepatology, King Khalid Hospital, Najran, Saudi Arabia

**Keywords:** endoscopic vacuum therapy (EVT), Endo-sponge, anastomotic leak, Hartmann reversal, colorectal surgery, diverting colostomy

## Abstract

Anastomotic leakage after Hartmann reversal is a challenging complication with limited management options. We report a 27-year-old woman who developed a recurrent leak after reversal surgery. Instead of repeat laparotomy, she underwent combined endoscopic vacuum-assisted therapy and diverting colostomy. Serial sponge exchanges promoted cavity collapse, infection resolution, and granulation, achieving healing without permanent diversion. This case illustrates the role of endoscopic vacuum-assisted therapy as a minimally invasive salvage option for recurrent colorectal leaks, highlighting its value in preserving bowel continuity and reducing morbidity in complex surgical scenarios.

## INTRODUCTION

Colorectal cancer frequently presents as an emergency, and left-sided disease often requires Hartmann procedure for obstruction or perforation.^1–5^ Although reversal restores continuity, it carries substantial morbidity. Anastomotic leakage (AL), reported in 2%–24% of low anterior resections, is among the most feared complications due to its association with reoperation, sepsis, and impaired survival.^6–8^

Traditional management of AL has involved takedown of the anastomosis and permanent diversion.^9,10^ Endoluminal vacuum-assisted therapy (EVT) has emerged as a minimally invasive alternative, promoting cavity collapse and healing through negative pressure.^11–14^ We present a young patient with a recurrent leak after Hartmann reversal successfully managed with EVT and diversion, underscoring its role as an organ-preserving salvage strategy.

## CASE REPORT

Our patient, a 27-year-old woman, initially underwent a laparoscopic low anterior resection for rectosigmoid cancer. Seven days postoperatively, she developed an anastomotic leak, which required an emergent open Hartmann procedure. The leak was associated with sepsis, abdominal pain, and elevated inflammatory markers, and the decision to proceed with Hartmann procedure was made to achieve rapid control of the source of contamination and stabilize the patient. After a 7-month recovery period, during which her nutritional status improved and surveillance imaging confirmed no recurrence of malignancy, she was admitted for Hartmann reversal. Preoperative imaging and endoscopic evaluation confirmed no residual malignancy and showed a healthy rectal stump, making her an appropriate candidate for reversal. The reversal was conducted with an end-to-end stapled anastomosis, with a prophylactic drain placed close to the new anastomosis. Intraoperative findings showed a tension-free anastomosis and good vascularity, and the procedure was considered technically successful.

Initially, her postoperative course appeared promising. She was mobilized early, tolerated liquids on postoperative day 1, and had stable laboratory parameters. However, on day 4, she developed persistent vomiting and intolerance of oral intake, which raised early concerns for anastomotic compromise. Although her abdominal examination revealed only mild distension and no peritoneal signs, the clinical team remained cautious, initiating supportive measures and close monitoring. We observed that her symptoms gradually improved, and laboratory values normalized over the next few days. This temporary clinical improvement provided reassurance, and on day 11, the prophylactic drain was removed.

Unfortunately, this stabilization was short-lived. On day 12, a flexible endoscopic examination was performed due to renewed abdominal discomfort and suspicion of a leak. The endoscopy revealed a recurrent defect at the anastomotic site, measuring approximately 1 × 2 cm, with associated purulent discharge. The endoscopic view confirmed a cavity extending beyond the anastomosis, consistent with a clinically significant leak. This finding prompted a rapid and multidisciplinary reassessment of management. Unlike the initial leak, which had necessitated immediate surgical takedown and creation of an end stoma, the recurrent leak was approached with an organ-preserving philosophy. The surgical and endoscopic teams jointly decided that a combination of fecal diversion and endoluminal therapy offered the best chance of preserving the anastomosis and avoiding repeat laparotomy (Table 1).

**Table 1.** Timeline of clinical course

POD/Date	Intervention	Outcome
August 2024	Laparoscopic low anterior resection with colorectal anastomosis and drain	Initial success
POD 7	Open exploration with anastomosis takedown and Hartmann procedure	Source control
7-mo recovery	Nutritional optimization & surveillance imaging	No recurrence
March 2025 (Reversal, POD 0)	Hartmann reversal with stapled anastomosis	Initial success
POD 4	Vomiting, poor intake	Concern for leak
POD 11	Drain removed	Temporary stabilization
POD 12	Flexible endoscopy: 1 × 2-cm leak	EVT + diversion planned
POD 13	Creation of diverting transverse colostomy	Diversion achieved
POD 16	First Endo-sponge placement^a^	ESBL *Escherichia coli* → meropenem
POD 20, 27, 34	Serial sponge exchanges	Progressive cavity reduction
POD 24	Repeat culture	Switched to ertapenem
POD 35	Final endoscopy: cavity ∼1 cm; Endo-sponge removed	Contrast CT: no extravasation; discharged stable
6-wk follow-up (June 2025)	Rectal contrast study and sigmoidoscopy	Completely healed anastomosis
October 2025	Routine prestoma closure contrast study	No extravasation
November 2025	Preoperative contrast study & sigmoidoscopy	Healed anastomosis; cleared for closure
November 2025	Loop colostomy closure	Uncomplicated recovery

CT, computed tomography; ESBL, extended-spectrum β-lactamase; EVT, endoscopic vacuum therapy; LAR, low anterior resection; POD, postoperative day.

a Endo-sponge, B. Braun Surgical, Spain.

**Figure 1.**
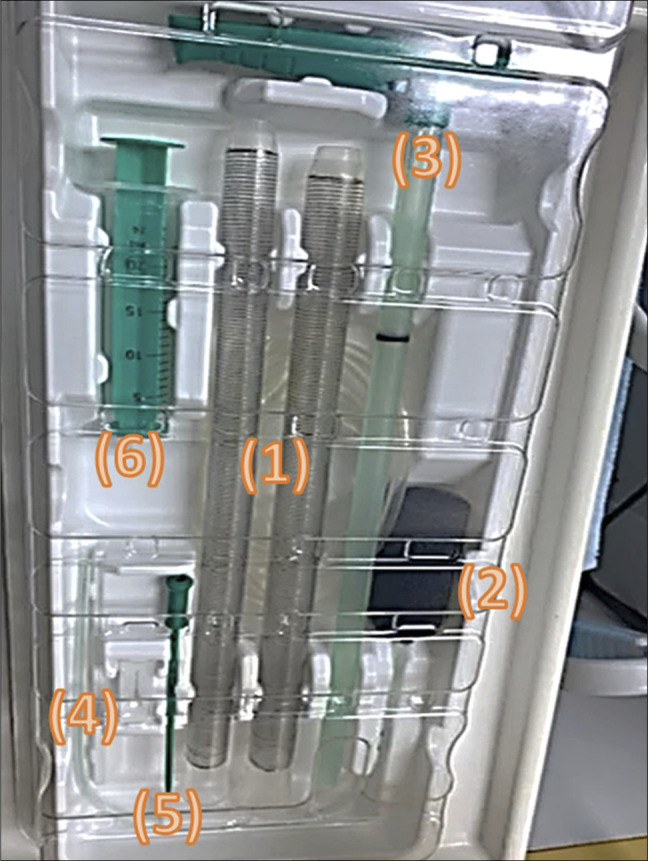
Components of the Endo-sponge kit: (1) Two sizes of overtubes used to deploy the Endo-sponge into the leakage cavity. (2) The Endo-sponge, designed to be placed within the cavity to absorb pus and leakage fluid while promoting approximation of the cavity edges. (3) The pusher, used to advance the Endo-sponge through the overtube to the leak site. (4) The catheter attached to the Endo-sponge, which allows for irrigation of the device. (5–6) Irrigation system, consisting of a syringe and a plastic connector, which can be attached to the catheter.

Management of the recurrent leak, therefore, involved 2 parallel interventions. First, a diverting transverse colostomy was created to defunction the anastomosis and divert the fecal stream. Second, an endoscopic vacuum-assisted drainage (Endo-sponge) device (B. Braun Surgical, S.A., Barcelona, Spain) was placed directly into the leak cavity under direct vision (Figure 1).

“… an Endo-sponge device was placed directly into the cavity (Figure 1).”

Placement was technically challenging because of the narrow pelvic space, but endoscopic guidance and fluoroscopic confirmation ensured accurate positioning. The sponge was connected to a vacuum system, applying continuous negative pressure.

**Figure 2.**
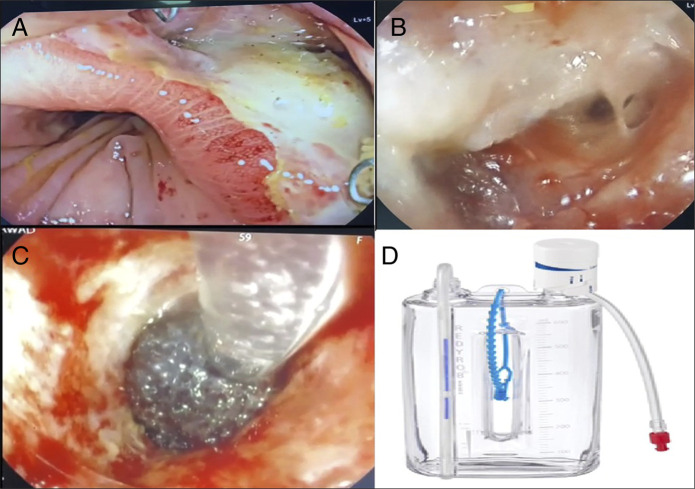
Endoscopic and device images during management of recurrent anastomotic leak. (A) Endoscopic view of the anastomotic site showing mucosal edema and inflammatory changes, with a defect through which the scope was advanced into the cavity. (B) Identification of the recurrent defect with purulent discharge. (C) Placement of the Endo-sponge into the leak cavity under direct endoscopic guidance. (D) Vacuum drainage bottle (REDYROB TRANS PLUS, Barcelona, Spain) connected to the Endo-sponge system, providing continuous negative pressure therapy.

Serial exchanges were performed under endoscopic control. The first exchange on postoperative day 16 demonstrated persistent purulent discharge, and cultures grew extended-spectrum β-lactamase–producing *Escherichia coli*. This organism was treated with intravenous meropenem, to which it was sensitive. The exchange procedure required careful removal of the previous sponge, which had become adherent to the granulating tissue. This was achieved with assistance from the surgical team using laparoscopic graspers under endoscopic guidance, minimizing trauma. Laparoscopic graspers were needed only for the first sponge exchange. Under endoscopic guidance, the graspers were passed via the anal verge to assist in freeing the sponge, which had become firmly adherent to early granulation tissue. All later exchanges were completed endoscopically without the need for adjunct instruments. A new Endo-sponge was then inserted, and the vacuum bottle (REDYROB TRANS PLUS, Barcelona, Spain) was connected, with suction pressure adjusted to promote granulation tissue formation and cavity collapse (Figure 2).

“…The system was connected to a vacuum drainage bottle to provide continuous negative pressure therapy (Figure 2).”

Subsequent exchanges on days 20, 27, and 34 showed steady improvement. Each procedure demonstrated progressive reduction of the cavity size, a cleaner wound field, and more robust granulation tissue. The surgical notes highlighted the absence of purulent discharge and the increasing resilience of the mucosal edges. On day 24, a repeat swab showed a shift in the bacterial resistance profile, necessitating a switch to ertapenem. This dynamic adjustment of antibiotic therapy ensured that systemic infection remained under control while local healing continued. Importantly, the patient tolerated all procedures well, with no episodes of hemodynamic instability, bleeding, or device dislodgement.

By day 35, the cavity had reduced to approximately 9–10 mm, with thick, healthy granulation tissue bridging the defect. At this point, the decision was made to remove the Endo-sponge permanently. Endoscopic inspection confirmed near-complete closure of the defect, and the patient was deemed safe for discharge. After final sponge removal, a water-soluble rectal contrast computed tomography was performed to confirm cavity collapse and absence of extraluminal leak. No contrast extravasation was seen.

At the time of discharge, she was tolerating a regular diet, mobilizing independently, and had stable laboratory parameters. She was subsequently referred back to the general surgical team for follow-up of the diverting colostomy and surveillance of the anastomosis. At her 6-week outpatient visit, she remained clinically stable, with no evidence of recurrent sepsis or leak, and her stoma was functioning well. Interval contrast studies in October and November 2025 were performed as part of routine surgical evaluation before stoma closure and demonstrated consistent absence of leak. After an uneventful recovery period of 8 months, she was readmitted under the general surgery service for planned stoma reversal. Preoperative flexible sigmoidoscopy demonstrated a healthy anastomotic site with intact mucosa and a normal vascular pattern, without evidence of residual defect or inflammation. A loop colostomy closure was subsequently performed without complication, and the patient was discharged in good condition.

**Figure 3.**
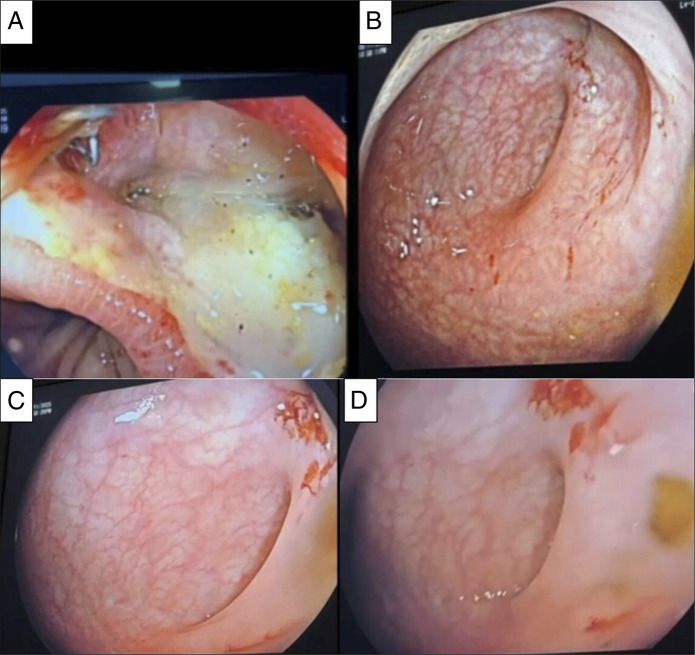
Follow-up endoscopic evaluation demonstrating complete healing of the anastomotic defect. (A) Initial endoscopic view showing the anastomotic defect with mucosal edema and exposed cavity. (B) Post-treatment view at 6-week follow-up demonstrating complete epithelialization of the defect. (C) Follow-up endoscopy showing a healthy anastomotic site with normal vascular pattern and no residual cavity. (D) Prestoma closure endoscopic view confirming intact mucosa and fully healed anastomosis.

“Follow-up endoscopy confirmed complete healing of the defect (Figure 3

).”

This complex clinical journey illustrates the role of combining endoscopic vacuum-assisted therapy with diversion in salvaging a recurrent anastomotic leak, preserving bowel continuity, and achieving resolution without resorting to permanent diversion.

## DISCUSSION

This case demonstrates 2 important lessons: (i) recurrent leakage can occur even after technically sound Hartmann reversal and (ii) EVT with diversion is an effective organ-preserving salvage approach.

Hartmann reversal is a high-risk operation, with morbidity rates exceeding elective colectomy.^5,10^ Although AL incidence ranges from 2% to 24%, recurrence after reversal is less reported.^6–8,15–18^ Historically, management required resection and permanent stoma, but this severely affects quality of life.^9,10,19,20^

EVT, introduced by Weidenhagen et al, actively drains cavities and stimulates healing.^11,13,14^ Multicenter series report closure rates of 60%–100%.^13,14^ Unlike stenting, EVT allows repeated inspection and remodeling of the cavity. When combined with diversion, as in our patient, EVT is especially effective for larger or recurrent leaks.

Our patient's resistant microbiology highlights the need for tailored antibiotics alongside EVT. Early initiation, within 2 weeks of detection, likely contributed to success. Reported complications include bleeding, sponge dislodgement, and chronic sinuses, but none occurred here.

Compared with prior literature, this case illustrates EVT's utility in recurrent leaks post-Hartmann reversal, a rare but clinically significant scenario. EVT allowed healing, preserved the anastomosis, and avoided repeat laparotomy.

**KEY CLINICAL TEACHING POINTS**

- Recurrent leakage may occur even after technically successful Hartmann reversal.
- Endoscopic reassessment is crucial for detecting subtle leaks.
- EVT provides active drainage, cavity remodeling, and granulation while preserving continuity.
- Combining EVT with diversion yields reliable healing in complex or recurrent leaks.

## DISCLOSURES

Author contributions: AG Al Masad and OR Abdelmaksoud contributed to management and drafting. AG Al Masad was primary endoscopist. All authors approved the final manuscript. OR Abdelmaksoud is the article guarantor.

Financial disclosure: None to report.

Informed consent was obtained for this case report.

AI disclosure: AI-assisted language editing (Chat GPT5) was used to improve grammar and readability, with all content reviewed and verified by the authors for accuracy and integrity.

## ABBREVIATIONS:

AL: anastomotic leakage
CT: computed tomography
ESBL: extended-spectrum beta-lactamase
EVT: endoscopic vacuum therapy
LAR: low anterior resection
POD: postoperative day
